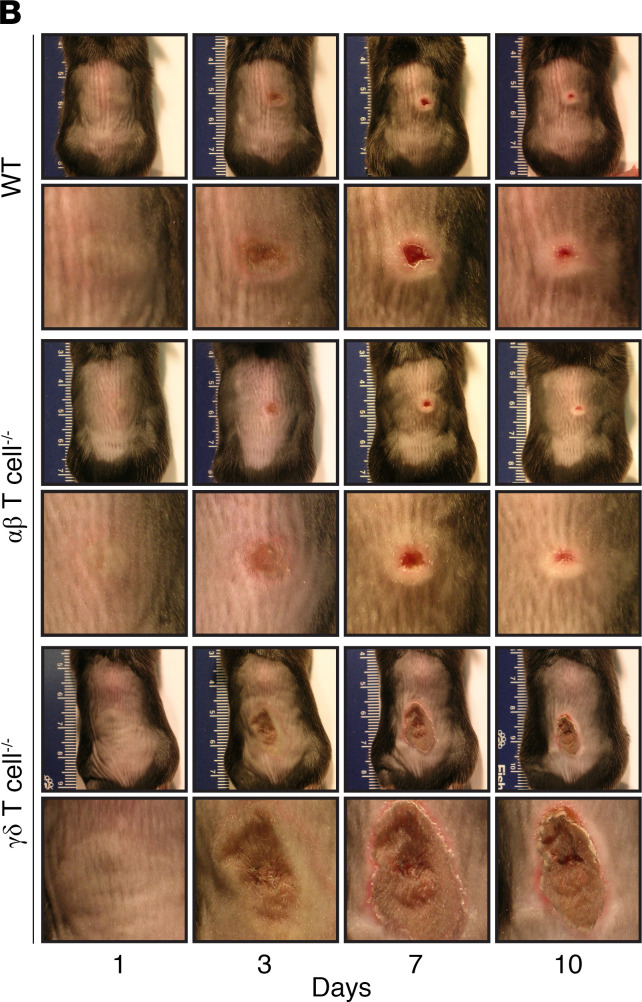# Corrigendum to IL-17 is essential for host defense against cutaneous *Staphylococcus aureus* infection in mice

**DOI:** 10.1172/JCI198106

**Published:** 2025-09-02

**Authors:** John S. Cho, Eric M. Pietras, Nairy C. Garcia, Romela Irene Ramos, David M. Farzam, Holly R. Monroe, Julie E. Magorien, Andrew Blauvelt, Jay K. Kolls, Ambrose L. Cheung, Genhong Cheng, Robert L. Modlin, Lloyd S. Miller

Original citation: *J Clin Invest*. 2010;120(5):1762–1773. https://doi.org/10.1172/JCI40891

Citation for this corrigendum: *J Clin Invest*. 2025;135(17):e198106. https://doi.org/10.1172/JCI198106

The authors became aware that in Figure 1B, the representative images for αβ T cell^–/–^ (row 4, column 1 and row 3, column 2) were inadvertently duplicated from the representative images for WT (row 2, column 1, and row 1, column 2, respectively) and the representative image for WT (row 2, column 2) and representative image for αβ T cell^–/–^ (row 4, column 2) were inadvertently interchanged. In addition, in Supplemental Figure 5, an incorrect image was used for the IL-1R^–/–^ sample. The supplemental material has been updated with the correct Supplemental Figure 5A. The correct Figure 1B provided from the original source data, is shown below.

The authors regret the errors.

## Supplementary Material

Supplemental data

## Figures and Tables

**Figure 1B:**