# Supplementary material for: Corrigendum to IL-17 is essential for host defense against cutaneous Staphylococcus aureus infection in mice
Source: J Clin Invest. 2025 Sep 2;135(17):e198106. doi: 10.1172/JCI198106 (PMC12404742; doi:10.1172/JCI198106)
Supplement: Supplemental data [file jci-135-198106-s002.pdf]

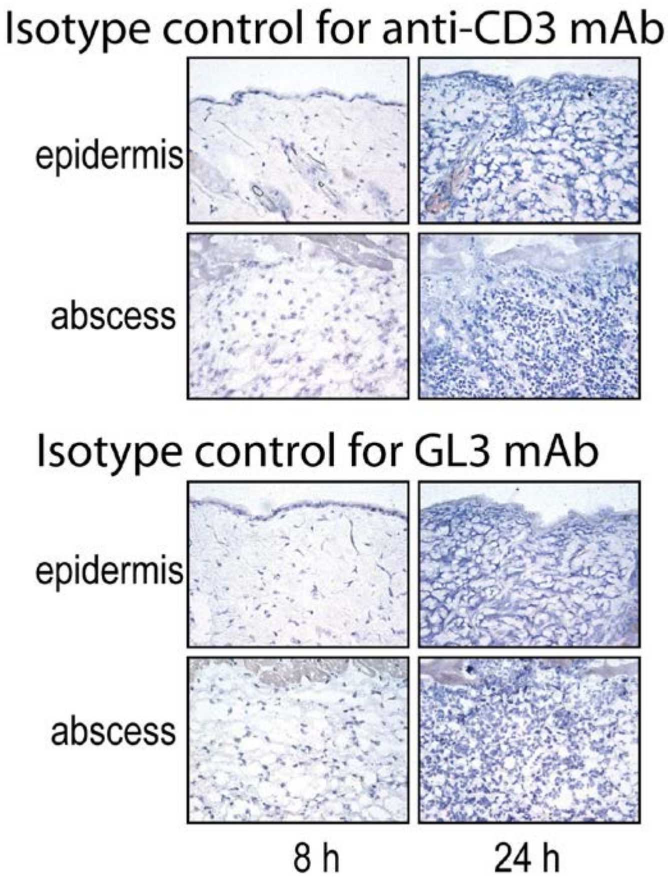

Supplemental Figure 1. Isotype controls for representative photomicrographs of histologic sections of wt mice after *S. aureus* infection presented in Figure 4. (A) Representative photomicrographs (400x) labeled with isotype control mAbs for CD3 (upper panels) and γδ T cells (GL3) (lower panels) (immunoperoxidase method) of histologic sections from skin biopsies from wt mice performed at 8 and 24 h after skin inoculation with *S. aureus* ($2x10^6$ CFUs in 100 μl PBS). There were no positively-labeled cells in any of the isotype control histologic sections for either the anti-CD3 mAb or the GL3 mAb.

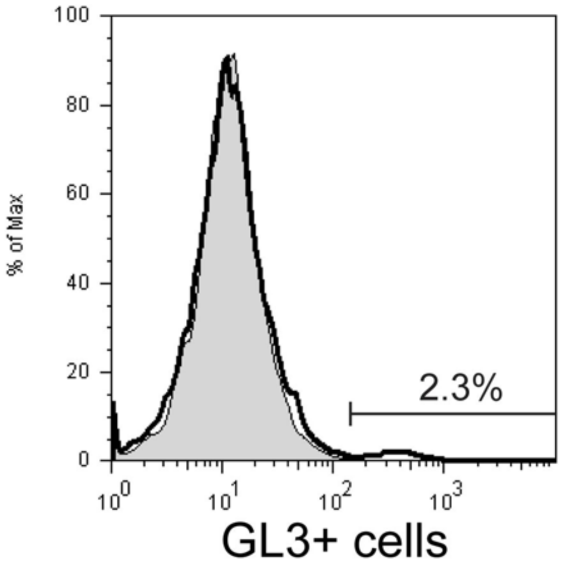

Supplemental Figure 2. Total proportion of γδ T cells in skin. Normal mouse skin was digested with collagenase (1 mg/ml) for 1 h at 37°C. Dead cells were removed by density gradient separation (Lympholyte M). Recovered live cells were labeled by using a γδ T cell specific mAb (clone GL3) (dark black line) or an isotype control mAb (gray, shaded) and analyzed by flow cytometry. γδ T cells represented 2.3% of total cells isolated from mouse epidermis. Data are representative from 2 experiments with at least 3 mice per experiment.

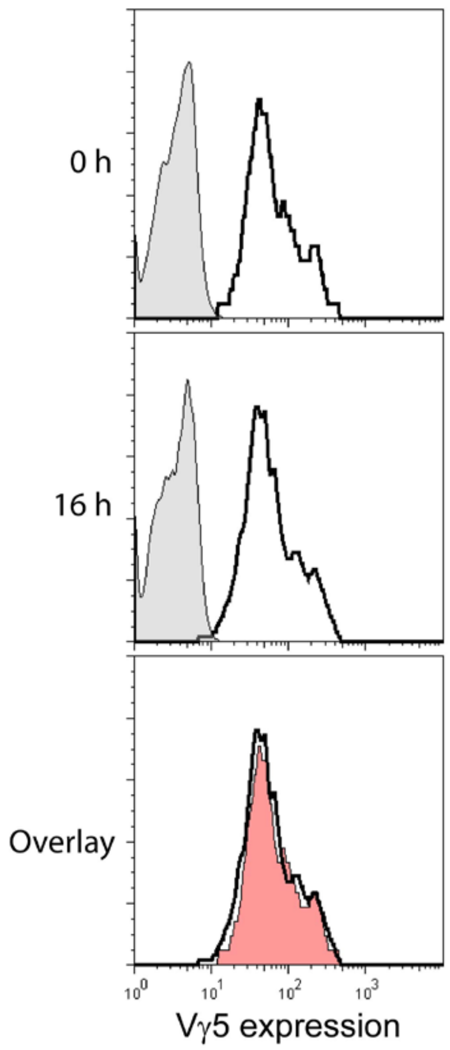

Supplemental Figure 3. Expression of Vγ5 TCR on γδ T cells after *S. aureus* cutaneous infection. Epidermal cell suspensions were harvested from wt mice at 0 h (uninfected skin) (top panel) and 16 h after *S. aureus* cutaneous inoculation (middle panel) and Vγ5 expression on epidermal γδ T cells (gated on $GL3^+$ cells) was determined by using a Vγ5-specific mAb or isotype control antibody (gray, shaded) and analyzed by flow cytometry. There was no difference in the Vγ5 expression at 0 and 16 h after infection as demonstrated in the bottom panel, which is an overlay of the expression of Vγ5 at 0 (red, shaded) and 16 h (dark black line) after cutaneous infection with *S. aureus*. Data are representative of mouse epidermal specimens from 6 wt mice pooled together at each time point.

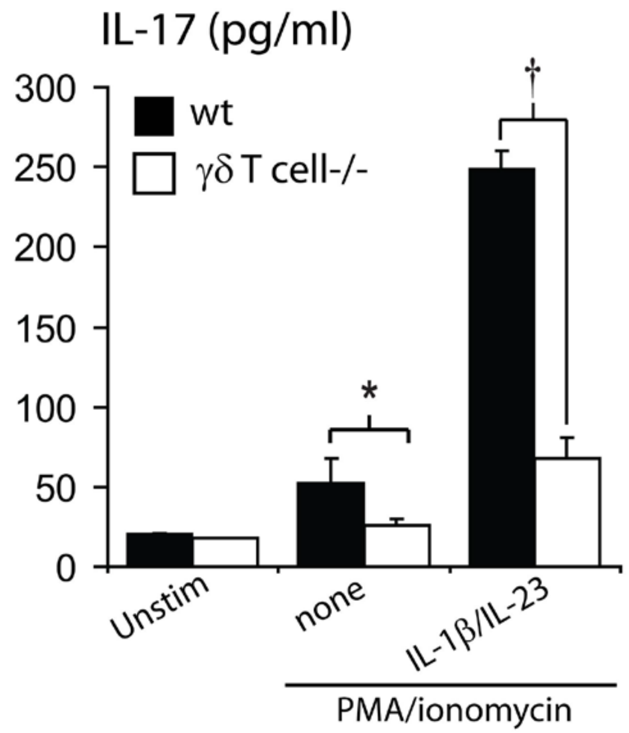

Supplemental Figure 4. Purified CD3$^+$ epidermal T cells (5x10^5 cells/ml) from normal skin of wt and γδ T cell-deficient mice were activated with PMA/ionomycin ± IL-1β/IL-23 (20 ng/ml of each). Supernatants were collected after 24 h for analysis of IL-17 protein (pg/ml) by ELISA (n=5/group). CD3$^+$ T cells isolated from γδ T cell-deficient mice produced substantially less IL-17 when compared with the CD3$^+$ T cells isolated from wt mice, which are comprised of mostly (~90%) γδ T cells (Figure 4B). This finding further demonstrates that the major population of cells responsible for IL-17 production after *S. aureus* infection is epidermal γδ T cells and not other compensatory T cells that present in the skin of γδ T cell-deficient mice.

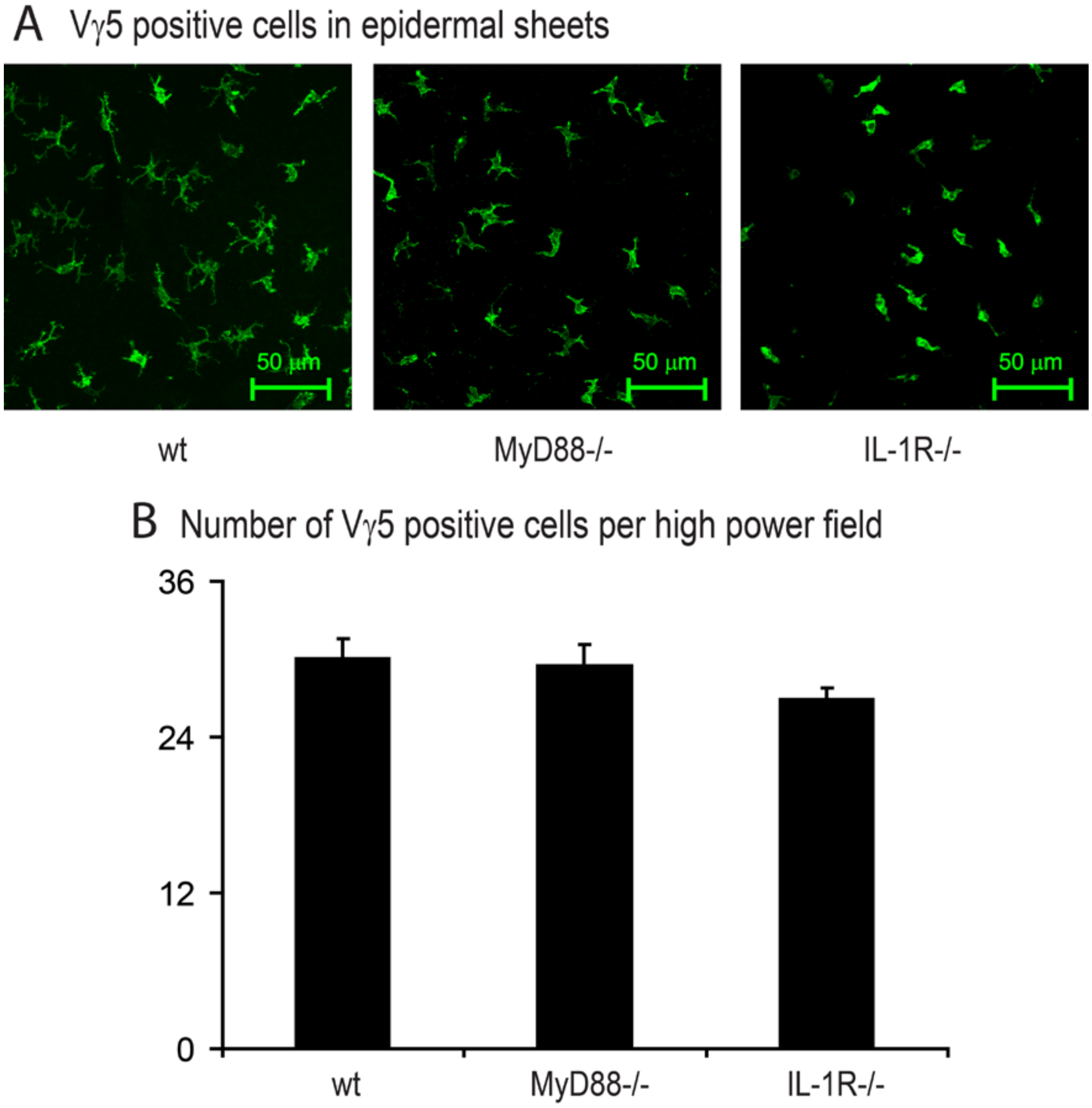

Supplemental Figure 5. Numbers of Vγ5 positive dendritic epidermal γδ T cells in the epidermis of wt, MyD88$^{-/-}$ and IL-1R$^{-/-}$ mice. (A) Representative confocal microscopy images of epidermal sheets from wt, MyD88$^{-/-}$ and IL-1R$^{-/-}$ mice that were labeled with an anti-Vγ5 mAb followed by an AlexaFluor488®-conjugated secondary antibody (630x magnification). (B) Mean numbers of Vγ5 cells per high power field ± SEM. Data represent the counts of Vγ5^{+} cells in 5 high power fields per specimen from 5 mice per group. There was no statistical difference in the number of Vγ5 positive dendritic epidermal γδ T cells in the epidermis of wt, MyD88$^{-/-}$ and IL-1R$^{-/-}$ mice.

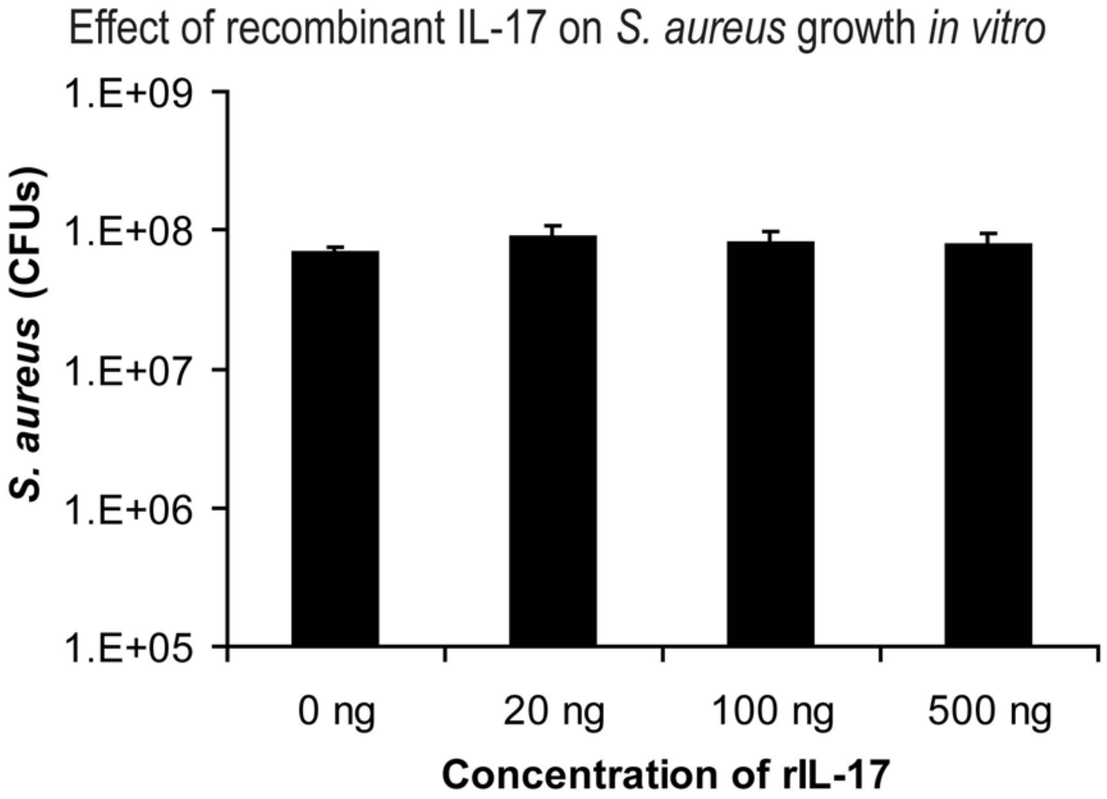

Supplemental Figure 6. To evaluate any potential bactericidal or bacteriostatic activity of IL-17 on *S. aureus*, we cultured *S. aureus* in shaking RPMI + 10% FCS for 5 h at 37°C in the presence increasing concentrations of recombinant murine IL-17 (rIL-17) (20, 100 and 500 ng) and numbers of *S. aureus* CFUs were determined after overnight culture on plates. We found that rIL-17 had no bactericidal or bacteriostatic activity against *S. aureus*.
